# Primary B-cell lymphoma of the renal pelvis following robotic nephroureterectomy: a case report

**DOI:** 10.1186/s12894-025-01968-4

**Published:** 2025-11-10

**Authors:** Thomas McMaster, Gabrielle Hoskin, Thomas Neerhut, Lauren Malacria, Matthew Farag, Luke Wang

**Affiliations:** 1 Sunshine Coast University Hospital, 6 Doherty Street, Birtinya, QLD 4575 Australia; 2 St Vincent’s Hospital Melbourne, 41 Victoria Parade, Fitzroy, VIC 3065 Australia

**Keywords:** Primary renal lymphoma, Urothelial, Robotic, Nephroureterectomy, Biopsy

## Abstract

**Background:**

We present a rare case of Primary Renal Lymphoma (PRL) diagnosed on histopathology following robotic nephroureterectomy. This case explores the diagnostic and management challenges urologists face, when treating renal masses.

**Case presentation:**

An 82-year-old male presented with recurrent macroscopic haematuria and a computed tomography intravenous pyelogram (CT IVP) reported renal papillary necrosis and an associated mass effect at the right renal pelvis. Urine cytology was inconclusive, whilst inpatient pyeloscopy revealed a renal pelvis tumour. This was biopsied and reported as suspicious for urothelial carcinoma. Multidisciplinary team discussion (MDT) recommended curative surgery. Following right nephroureterectomy, postoperative histology revealed a diffuse large B-Cell lymphoma (DLBCL) extending through the renal capsule into perirenal fat, renal vein and renal pelvis.

**Conclusion:**

We highlight the clinical similarities between upper tract urothelial carcinoma (UTUC) and PRL, encouraging urologists to consider biopsies of indeterminate lesions, particularly amongst the frail population.

## Background

Primary Renal Lymphoma (PRL) is an exceptionally rare and aggressive form of extra-nodal non-Hodgkin’s lymphoma (NHL), accounting for less than 1% of all renal masses [1]. Its origin in the kidney is unusual due to the absence of native lymphoid tissue, and its pathogenesis remains poorly understood [1]. Owing to its rarity, published literature is limited and diagnosis is frequently missed or delayed. PRL often mimics more common renal malignancies such as Renal Cell Carcinoma (RCC) or upper tract urothelial carcinoma (UTUC), leading to unnecessary surgical interventions and delayed systemic therapy [2–4]. Accurate histopathological diagnosis, typically via renal biopsy, is essential for appropriate management and improved outcomes.

We report a rare case of PRL, identified post-operatively following robotic nephroureterectomy. This work has been reported in line with the CARE guidelines [5].

## Case presentation

**Fig. 1:**
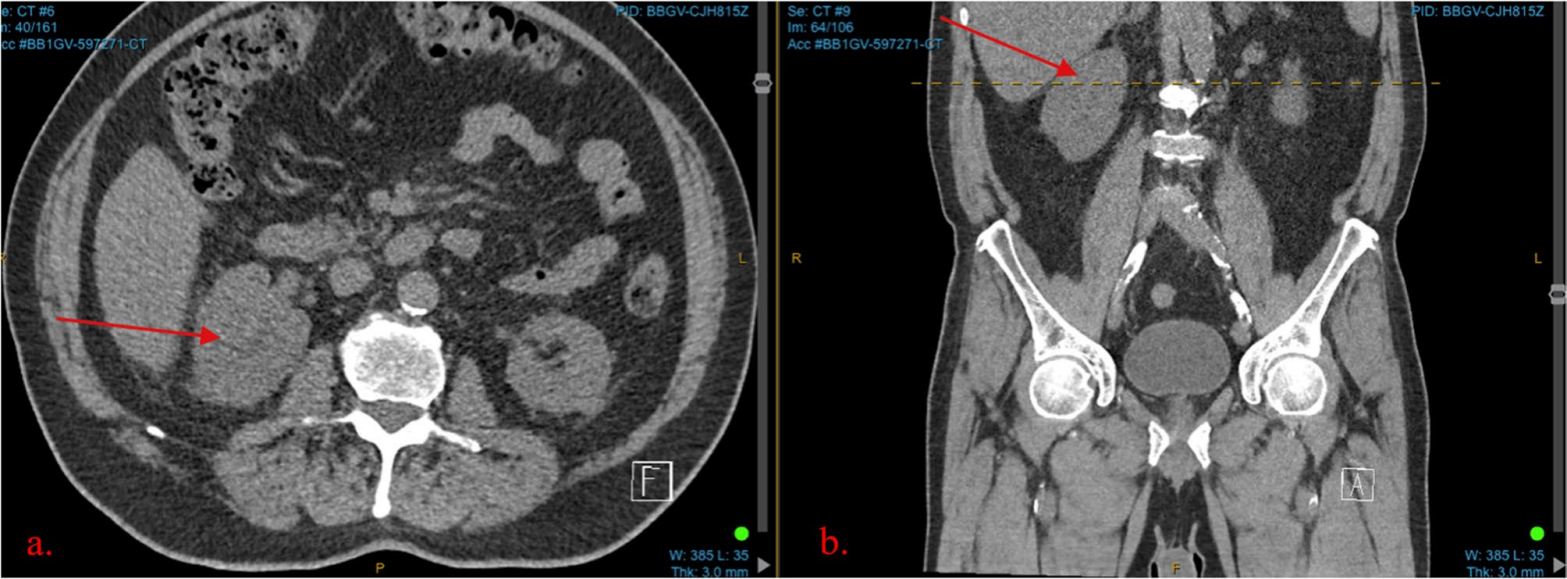
Non-contrast CT Kidney, ureter and bladder. **a ** An axial view demonstrating loss of clarity of right upper pole renal sinus. **b** A coronal view demonstrating the same

**Fig. 2:**
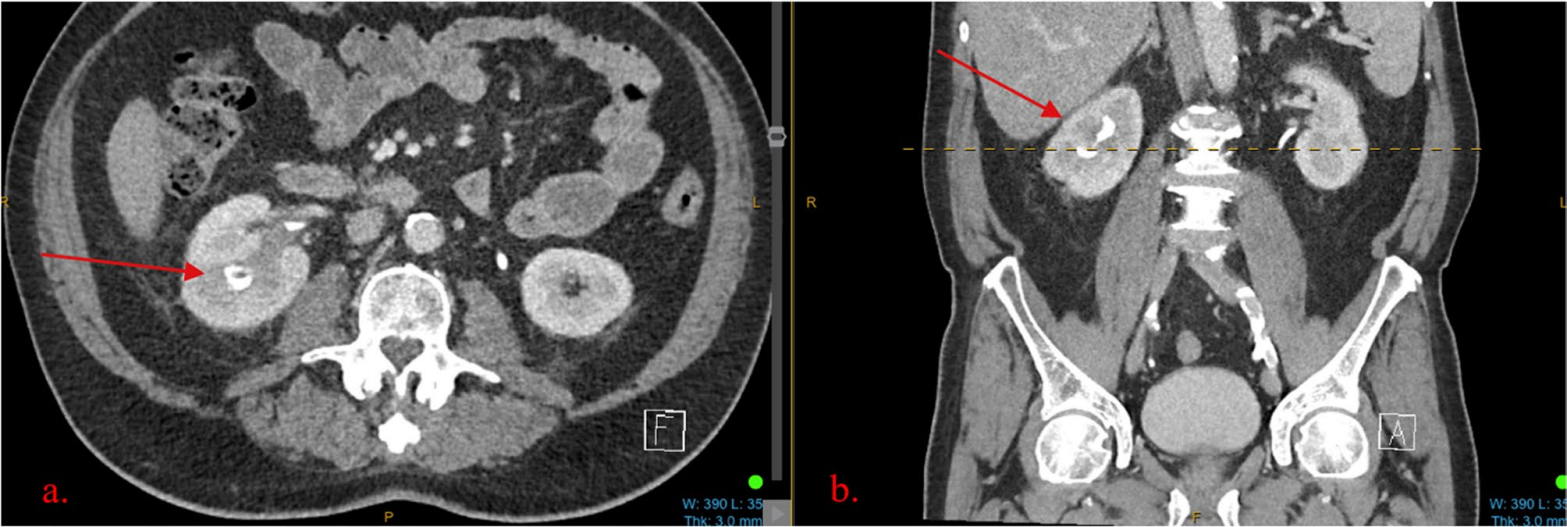
CT Intravenous pyelogram. **a** An axial view demonstrating renal papillary necrosis at the right upper pole with underfilling, associated mass effect at the renal pelvis. **b** A coronal view demonstrating the same

An 82-year-old man presented with recurrent macroscopic haematuria and right flank pain. A non-contrast computed tomography (CT) scan was initially ordered, reporting a loss of clarity of the right upper pole renal sinus (see Fig. 1). A follow up CT intravenous pyelogram (CT IVP) reported renal papillary necrosis at the right upper pole with associated mass effect at the renal pelvis and appearances suggestive of parapelvic cysts (see Fig. 2). CT chest was unremarkable and urine cytology was negative for high grade urothelial carcinoma. Given the radiological findings, an inpatient pyeloscopy was performed, revealing a macroscopic lesion of the renal pelvis. This was biopsied at the time; however, the sample was deemed insufficient for conclusive histopathological analysis. Clusters of atypical epithelioid cells without unequivocal features of malignancy were noted. This was later verified by another pathologist who reported the sample as suspicious for urothelial carcinoma. The case was discussed at a multi-disciplinary team (MDT) meeting, noting that during pyeloscopy, the lesion was observed to be a tumour consistent with the typical macroscopic appearance of high grade invasive primary urothelial carcinoma.

Following the MDT, the patient was consented and booked for a right robotic nephroureterectomy, with peri-operative medicine and anaesthetic review prior. He was deemed a high-risk surgical candidate, with comorbidities including ischaemic heart disease, diabetes and hypertension, however, cardiac investigations did not preclude surgery. The robotic assisted procedure was uncomplicated, however, in the post-operative period, the patient suffered a type two myocardial infarction, that was conservatively managed.

Despite a presumed diagnosis of invasive upper tract urothelial carcinoma, histopathology revealed a Diffuse Large B-Cell Lymphoma (DLBCL), extending through the renal capsule into perirenal fat, renal vein and renal pelvis. The patient was then referred to the haematology team, who discussed both the role of formal staging with Positron Emission Tomography (PET) imaging as well as the rationale for chemotherapy. After sufficient counselling, the patient declined all further investigations and management, instead, opting for palliation and a referral for consideration of voluntary assisted dying.

## Discussion

PRL is a rare diagnosis, accounting for less than 1% of renal masses [1]. Its clinical and radiological overlap with UTUC makes differentiation challenging, often leading to misdiagnosis and unnecessary surgery. This is particularly significant in elderly or comorbid patients, where avoiding invasive procedures may improve outcomes.

### Epidemiology

PRL shows a slight male predominance and occurs more frequently in white, middle-aged to elderly individuals [4]. UTUC, similarly more common in older men, is strongly associated with tobacco exposure and peaks between ages 70–90 [6].

### Clinical presentation

PRL often presents unilaterally and is asymptomatic in up to 50% of cases [1]. When symptomatic, younger patients tend to report flank pain, whilst older individuals may present with haematuria or weight loss [4]. Constitutional features of lymphoma, such as, fever or elevated LDH may also be seen. UTUC commonly presents with haematuria and flank pain, symptoms that also correlate with disease progression [6].

### Investigation

#### Imaging

CT remains the mainstay radiological investigation for both PRL and UTUC. PRL may appear as a solitary or multifocal mass, with or without nodal involvement, and sometimes demonstrates atypical features like necrosis or infiltration [7]. 18 F-FDG PET CT may aid diagnosis and staging [7]. CT urography offers high diagnostic accuracy for UTUC, though distinguishing it from other renal masses (e.g., renal cell carcinoma (RCC), other metastases, focal xanthogranulomatous pyelonephritis (XGP), or PRL) can be difficult, particularly when the renal architecture is distorted [3, 8].

#### Diagnostic procedures

When imaging and urine cytology are inconclusive, diagnostic ureteroscopy (dURS) with biopsy allows visualisation and sampling of upper tract lesions [8]. Although dURS carries a potential risk of tumour seeding, it remains a valuable tool in selecting appropriate management pathways.

### Management

Due to its rarity, there is no standardised treatment for PRL, but systemic chemoimmunotherapy agents, such as R-CHOP (rituximab, cyclophosphamide, vincristine, prednisolone), remain the cornerstone of B cell PRL treatment [9]. Some reports suggest benefit from surgical resection followed by adjuvant therapy [10], though evidence is limited. Conversely, UTUC management is well-established and guided by risk stratification: low-risk disease may be managed conservatively, while high-risk cases typically require radical nephroureterectomy and systemic therapy [9].

## Conclusion

We have reported a rare case of PRL diagnosed on histopathology following robotic nephroureterectomy. PRL is a rare and often misdiagnosed renal malignancy, easily mistaken for more common pathologies such as UTUC. This case underscores the importance of physicians maintaining a high index of suspicion for rare pathologies, particularly when standard investigations are inconclusive or clinical presentations are atypical.

## Data Availability

Medical records pertaining to the case report may be found on the electronic medical records system at St Vincent’s Hospital Melbourne, VIC Australia.

## Abbreviations

PRL: Primary Renal Lymphoma
CT IVP: Computed Tomography Intravenous Pyelogram
UTUC: Upper Tract Urothelial Carcinoma
MDT: Multidisciplinary team
NHL: Non-Hodgkin’s Lymphoma
PET: Positron Emission Tomography
RCC: Renal Cell Carcinoma
DLBCL: Diffuse Large B-Cell Lymphoma
XGP: Xanthogranulomatous Pyelonephritis
dURS: Diagnostic Ureteroscopy
R-CHOP: Rituximab, Cyclophosphamide, Vincristine, Prednisolone
